# Urinary cGMP concentrations after exposure to radio-contrast media in patient at risk for contrast media induced nephropathy (CIN) predicts 90-day morbidity and mortality

**DOI:** 10.1186/2050-6511-14-S1-P29

**Published:** 2013-08-29

**Authors:** Berthold Hocher, Fabian Heunisch, Gina-Franziska von Einem, Markus Alter, Yvonne Keim, Johannes-Peter Stasch

**Affiliations:** 1 Institute for Nutritional Science, University of Potsdam, Potsdam, Germany; 2 Center for Cardiovascular Research, Charité, Berlin, Germany; 3 Bayer Pharma AG, Wuppertal, Germany

## Background

Acute renal failure after exposure to contrast media in patients at risk for CIN increases substantially 90-day mortality and morbidity. Biomarkers for outcome after CIN are missing so far.

## Results

We analyzed in a prospective study 233 patients with either diabetes or preexisting impaired kidney function getting intra-arterial contrast media (CM) for coronary angiograms. Urinary cGMP concentrations were analyzed 24 hours after exposure to intra-arterial CM in spot urine. The patients were followed for 90 day for the following events: death, need for transient or permanent dialysis, doubling of serum creatinine and unplanned re-hospitalization. 51 out of the 233 patients had such an event during the follow up period of 90 days after contrast media exposure. The urinary cGMP to creatinine ratio was significantly increased in patients having an event during the follow up period (p=0.011). We furthermore performed a multivariate regression analysis considering age, baseline kidney function before contrast media exposure and preexisting diabetes - all important risk factors for CIN. These analysis revealed that the urinary cGMP to creatinine ratio is an in dependent predictor of the occurrence of the composite endpoint: death, need for transient or permanent dialysis, doubling of serum creatinine and unplanned re-hospitalization (p=0.010; B=0.001; T=2.584; CI= 0.001 – 0.003).

## Conclusion

The urinary cGMP to creatinineratio isan independent predictor of major advers events after contrast media exposure during a follow-up of 90 days.

